# Photoacoustic monitoring of tumor and normal tissue response to radiation

**DOI:** 10.1038/srep21237

**Published:** 2016-02-17

**Authors:** Laurie J. Rich, Mukund Seshadri

**Affiliations:** 1 Departments of Molecular and Cellular Biophysics and Biochemistry, Roswell Park Cancer Institute, Buffalo, NY 14263; 2 Departments of Pharmacology and Therapeutics, Roswell Park Cancer Institute, Buffalo, NY 14263; 3 Oral Medicine/Head and Neck Surgery, Roswell Park Cancer Institute, Buffalo, NY 14263.

## Abstract

Hypoxia is a recognized characteristic of tumors that influences efficacy of radiotherapy (RT). Photoacoustic imaging (PAI) is a relatively new imaging technique that exploits the optical characteristics of hemoglobin to provide information on tissue oxygenation. In the present study, PAI based measures of tumor oxygen saturation (%sO_2_) were compared to oxygen-enhanced magnetic resonance imaging (MRI) measurements of longitudinal relaxation rate (R1 = 1/T1) and *ex-vivo* histology in patient derived xenograft (PDX) models of head and neck cancer. PAI was utilized to assess early changes (24 h) in %sO_2_ following RT and chemoRT (CRT) and to assess changes in salivary gland hemodynamics following radiation. A significant increase in tumor %sO_2_ and R1 was observed following oxygen inhalation. Good spatial correlation was observed between PAI, MRI and histology. An early increase in %sO_2_ after RT and CRT detected by PAI was associated with significant tumor growth inhibition. Twenty four hours after RT, PAI also detected loss of hemodynamic response to gustatory stimulation in murine salivary gland tissue suggestive of radiation-induced vascular damage. Our observations illustrate the utility of PAI in detecting tumor and normal tissue hemodynamic response to radiation in head and neck cancers.

Hypoxia is a major cause of resistance to radiotherapy (RT) and a well-documented prognostic factor in solid tumors including head and neck squamous cell carcinomas (HNSCC)^1^,^2^. Yet, no single method to measure tumor oxygenation status has been established for clinical use. Early studies of hypoxia involved the use of oxygen-sensitive polarographic electrodes to measure the partial pressure of oxygen (pO_2_) in tumors^2^,^3^. While this method is sensitive, it is invasive and limited in providing spatial information on oxygenation levels throughout the tumor. Additionally, its invasive nature prevents serial measurements of tumor oxygenation throughout the course of treatment. In contrast, non-invasive imaging methods offer the potential to longitudinally monitor dynamic temporal changes occurring within the tumor microenvironment and allow us to map the spatial heterogeneity in tumor oxygenation^4^. Conventional imaging methods such as magnetic resonance imaging (MRI), positron emission tomography (PET) and computed tomography (CT) have been utilized to study tumor vascularity and oxygenation in animal models and in patients^5^,^6^. However, these technologies are expensive and require the use of ionizing radiation or radioisotopes. Development of a cheap, reliable and easy to use imaging method to image tumor oxygenation would therefore be of immense clinical value.

Photoacoustic imaging (PAI) is a hybrid imaging technique that combines the sensitivity of optical imaging and the resolution of ultrasound (US)^7^,^8^,^9^. In PAI, tissue is illuminated with light (ranging from 700–900 nm in wavelength), which is absorbed by endogenous chromophores such as hemoglobin (Hb) resulting in thermo-elastic expansion and generation of a broadband pressure wave that is detected as an acoustic signal^7^,^8^,^9^. Interestingly, the absorption characteristics of Hb are influenced by whether it is oxygen-bound (oxy-hemoglobin; absorption peak at 850 nm) or free (deoxy-hemoglobin; absorption peak 750 nm)^10^. Comparing the PA signal at these two wavelengths allows for calculation of oxygen saturation (%sO_2_) of blood while the sum of the two signals provides a measure of total Hb concentration in tissue^10^,^11^,^12^. In this manner, PAI exploits endogenous contrast mechanisms to enable visualization of tissue microvascular structure and hemodynamics. As a result, there has been widespread interest in the development of PAI for medical applications, particularly in cancer research^13^,^14^. Studies in preclinical models have demonstrated the utility of PAI for monitoring tumor response to photodynamic therapy and vascular-targeted therapies^15^,^16^. However, the prognostic utility of PAI for monitoring tumor oxygenation in response to RT in HNSCC has not been previously examined.

Given the influence of oxygenation on radiotherapeutic efficacy, we hypothesized that assessment of early changes in tumor oxygenation using PAI could serve as a biomarker of RT response in HNSCC. To test this hypothesis, experimental PAI was performed in patient-derived xenograft (PDX) models of HNSCC to assess acute changes in tumor oxygenation following radiation. Studies were also conducted to examine the response of salivary glands, a normal tissue target that is often affected by RT in head and neck cancer patients. Our results demonstrate, for the first time, that PAI can detect early changes in oxygenation of tumor and normal tissues following radiation.

## Results

### Combined PAI and OE-MRI of oxygen dynamics in HNSCC

**Figure 1:**
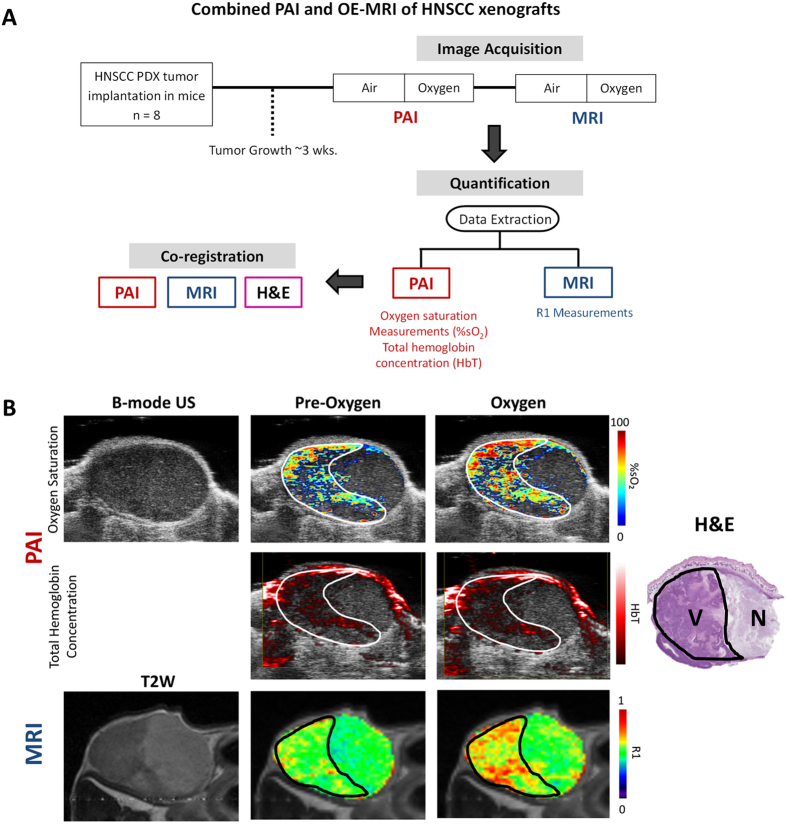
Combined PAI and OE-MRI of HNSCC hemodynamics *in vivo*. (**A**) Work flow for combined oxygen-enhanced PAI, MRI and histologic correlation of vascular hemodynamics in HNSCC xenografts. Tumor-bearing mice were exposed to a regimen of room air for 2 minutes followed by inhalation of 100% of oxygen (hyperoxia) for 6 minutes and subsequently returned to room air. (**B**) *Top row*: B-mode US and co-registered PA maps of oxygen saturation of PDX-HNSCC during exposure to room air (pre-oxygen) and following oxygen (100%; hyperoxia); *Middle row:* hemoglobin concentration maps of the same tumor before and after oxygen challenge; *Bottom row*: Axial T2-weighted image (left) and longitudinal relaxation rate (R1) color maps of the tumor under the same conditions. Corresponding histologic section of the tumor (H&E) is shown on the right. Response to oxygen challenge was localized to the viable (V) region of the tumor. No response was detected on PAI or MRI in the necrotic region of the tumor (N).

**Figure 2:**
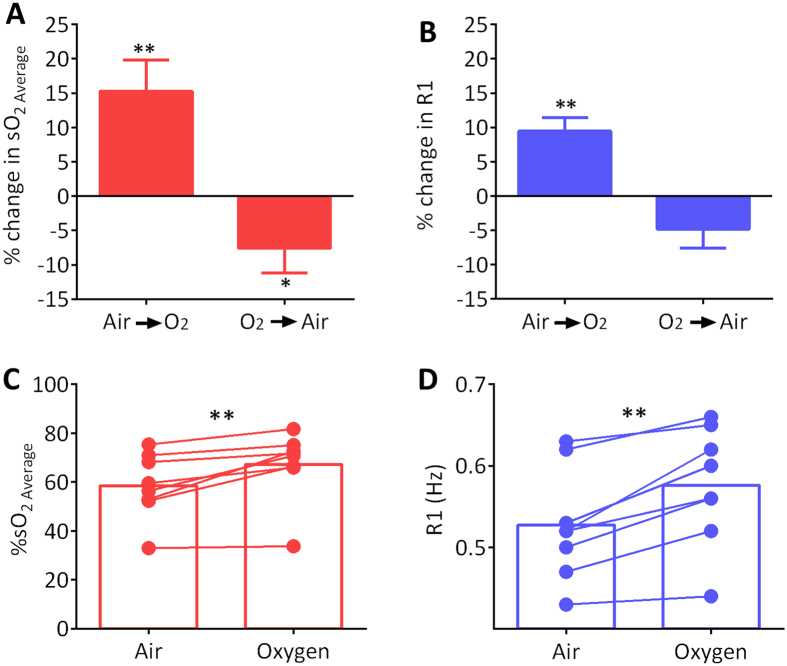
PAI and MRI of tumor response to hyperoxia. Bar graphs show relative change in %sO_2_ on PAI (**A**) and R1 on MRI (**B**) during transition from room air to oxygen (Air → O_2_) and from oxygen to room air (O_2_ → air). Values of %SO_2_ (**C**), R1 (**D**) of individual tumors before and after oxygen challenge are shown in the bottom. The reported %SO_2 Average_ values represent the average value from all pixels in the tumor with an oxygen saturation estimate excluding those with zero values/estimate void pixels. *denotes p < 0.05; **denotes p < 0.01

We first compared PAI measures of tumor oxygen saturation (%SO_2_) with oxygen-enhanced magnetic resonance imaging (OE-MRI), a non-invasive imaging technique that is currently being examined as a biomarker of tumor oxygenation in patients^17^. Experimental studies were conducted in PDX-HNSCC to examine tumor response to hyperoxia (inhalation of 100% oxygen) using PAI and OE-MRI. The relative change in oxygen saturation (%SO_2_) and longitudinal relaxation rate (R1 =1/T1) following hyperoxia were calculated from PAI and MRI datasets, respectively (Fig. 1A). Hyperoxic challenge led to a visible increase in %SO_2_ (Fig. 1B, *top panel*) and R1 (Fig. 1B, *bottom panel*), on PAI and MRI, respectively (Fig. 1B). Histologic evaluation of the tumor (Fig. 1B, H&E) revealed that the signal change observed on PAI (*outlined in white*) and MRI (*outlined in black*) was localized to the viable tumor region (V). No change in PAI or MR signal was detected in response to hyperoxia in the necrotic region of the tumor (N). Ex-vivo histology data for the remaining 7 tumors that underwent imaging is shown in Supplementary Fig. S1. Spatial alignment between MRI and PAI was not possible in one of the tumors (m4). While the degree of spatial correlation was limited in some of the tumors (m2, m5), we observed good correlation between PAI and histology in 6/8 tumors (m1, m3, m4, m5, m7, m8) and between PAI with OE-MRI in 5/7 tumors (m1, m3, m6, m7, m8) (Supplementary Fig. S1). Corresponding measures of total Hb concentration in the tumor did not show any change following oxygen inhalation (Fig. 1A, *middle panel* and Supplementary Fig. S2). Transitioning from room air to oxygen resulted in a significant increase in %SO_2_ (15.3 ± 4.5%, p = 0.005; Fig. 2A) and R1 (9.5 ± 1.9%, p = 0.002; Fig. 2B) as seen on PAI and MRI, respectively. A reduction in tumor %SO_2_ (7.5 ± 3.6%, p = 0.039) and R1 (4.8 ± 2.8%; p > 0.05) was also observed when transitioning back from oxygen to room air. Quantitative estimates of %SO_2_ (Fig. 2C) and R1 (Fig. 2D) illustrate the heterogeneity in the response of individual tumors to hyperoxic challenge. Whole tumor estimates did not reveal any correlation between the change in %SO_2_ and R1 of individual tumors following hyperoxia (Supplementary Fig. S3). However, median values of the post-oxygen PAI signal at 850 nm showed a positive correlation with median R1 values (Supplementary Fig. S3; p = 0.08). Region of interest (ROI) analysis of %SO_2_ and R1 following oxygen challenge showed a significant correlation between PAI and MRI (Supplementary Fig. S4). A significant correlation was observed between median PAI signal at 850 nm and percentage of viable tumor on histology (Supplementary Fig. S5).

### PAI allows early monitoring of tumor response to radiation and chemoradiation

**Figure 3:**
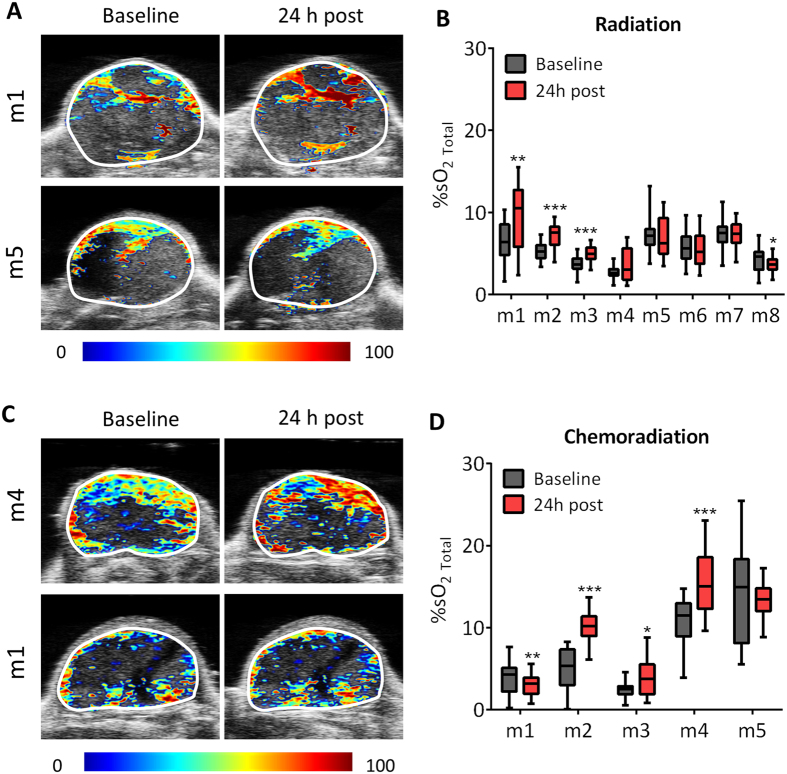
PAI allows early monitoring of tumor response to RT and CRT. The panel of images represent pseudo-colorized tumor oxygen saturation maps of individual tumors (two per treatment) overlaid on the B-mode ultrasound image at baseline and 24 h post RT (n = 8; A) or CRT (n = 5; C). Compared to baseline estimates, some tumors showed a positive change in oxygen saturation post treatment (Fig. A, C, *top panel*) while others showed minimal change following RT or CRT (Fig. A,C, *bottom panel*) Corresponding box-and-whisker plots of %SO_2 Total_ levels of individual tumors at baseline and 24 h post RT (**B**) and CRT (**D**) are also shown. Values of %SO_2 Total_ reported represent the average value calculated from all pixels including those with zero/estimate void pixels. Box-and-whiskers show min and max values (whiskers), 25^th^ to 75^th^ percentiles (box), and median %SO_2 Total._

Next, we examined if PAI could detect early changes in oxygenation following radiation *in vivo*. PAI examination was performed in tumor-bearing mice at baseline and 24 h post radiation alone or in combination with chemotherapy. The panel of images shown in Fig. 3A,C represents %SO_2_ maps overlaid on the B-mode ultrasound image of tumors at baseline and 24 h post RT and chemoRT (CRT), respectively. PAI demonstrated the heterogeneity in hemodynamic response of individual tumors to RT and CRT. Compared to baseline estimates, some tumors showed a positive change in oxygen saturation post treatment (Fig. 3A,C, *top panel*) while others showed minimal change following RT or CRT (Fig. 3A,C, *bottom panel*). Quantitative estimates of change in oxygen saturation of individual tumors following RT (Fig. 3B) and CRT (Fig. 3D) are also shown. PAI of tumor-bearing mice that underwent CRT also demonstrated inter-tumor variability in the magnitude and direction of change in oxygen saturation 24 hours after treatment (Fig. 3C,D).

### Early oxygenation change following radiation detected by PAI correlates with outcome

**Figure 4:**
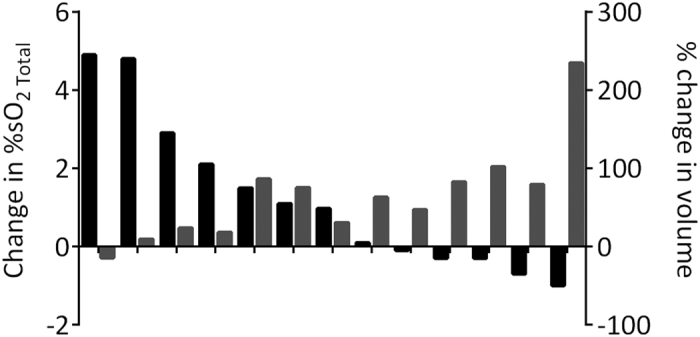
Early change in oxygenation following RT detected by PAI correlates with outcome. Waterfall plot showing individual changes in tumor %SO_2 Total_ at 24 h post treatment and change in tumor volume at 2 weeks post treatment. A significant correlation (r = −0.7191; p < 0.01) was observed between the percent change in %SO_2_ observed at 24 h and change in tumor volume measured at 2 weeks post therapy

It was our hypothesis that PAI could provide an early read-out of tumor response to RT prior to detectable change in tumor volume. To test this hypothesis, early treatment-induced changes in parameters of Hb concentration and %SO_2_ measured by PAI were correlated with the outcome of individual tumors. Figure 4 shows a waterfall plot comparing changes in %SO_2_ of individual tumors at 24 h post treatment and the corresponding change in tumor volume at 2 weeks post treatment. This analysis revealed that tumors that showed an early increase in %SO_2_ following RT or CRT responded more favorably (based on tumor growth inhibition at 2 weeks) than those that showed no change or a reduction in %SO_2_ on PAI examination at 24 h post treatment (Fig. 4). A significant correlation (r = −0.7191; p < 0.01) was observed between the percent change in %SO_2_ observed at 24 h and change in tumor volume measured at 2 weeks post therapy (Supplementary Fig. S6). No relationship was observed between the measured total Hb concentration and outcome. These results demonstrate that early oxygenation change following RT detected by PAI correlates with outcome.

### PAI of radiation-induced salivary gland injury

**Figure 5:**
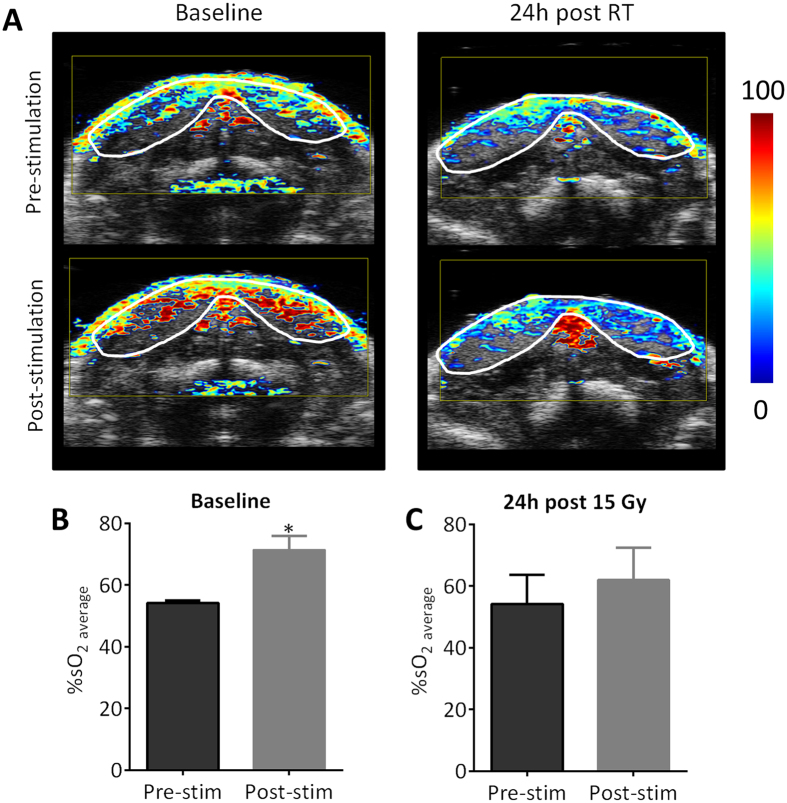
PAI of radiation-induced salivary gland injury in mice. (**A**) Parametric maps of %SO_2_ at baseline and 24 h post 15 Gy RT. Images were obtained before (pre-stimulation) and after (post-stimulation) gustatory stimulation using topical citric acid application. Oxygen saturation measurements of mouse salivary glands pre- and post-stimulation at baseline (**B**) and 24 h post 15 Gy RT (**C**) Hemodynamic response to stimulation was reduced 24 hour after RT suggestive of radiation-induced vascular damage.

Salivary glands are highly radio-sensitive organs and salivary gland dysfunction is a common and often severe side-effect of RT in patients with head and neck cancer^18^. Vascular damage is considered to be a major contributor of salivary gland dysfunction following RT^18^,^19^. We therefore conducted studies in mice to examine early changes in salivary gland hemodynamics following RT using PAI (Fig. 5). Baseline assessment (pre-RT) of salivary gland function was obtained in naïve mice (n = 4) before and after gustatory stimulation. Mouse salivary gland tissue was then exposed to 15 Gy and PAI was performed 24 h post treatment to assess early parenchymal and vascular damage following RT. Hemodynamic response to stimulation was visualized and quantified at baseline and 24 h post RT. The panel of images shown in Fig. 5A represents parametric maps of %SO_2_ of salivary glands obtained before and after gustatory stimulation at the two time points. A significant increase in %SO_2_ (54.2 ± 0.8 to 71.3 ± 4.5, p = 0.035) was observed at baseline following citric acid stimulation (Fig. 5B) reflective of the hemodynamic response associated with salivary secretion. In comparison, hemodynamic response to gustatory stimulation was lost 24 h following RT (Fig. 5C). No change in salivary gland HbT was observed in response to stimulation at baseline or following RT (Supplementary Fig. S7).

## Discussion

HNSCCs constitute a biologically heterogeneous group of neoplasms that can recur despite aggressive therapeutic intervention^20^. A consistent body of evidence exists regarding the impact of hypoxia on the RT response of HNSCC^1^,^2^,^21^. It was therefore our hypothesis that non-invasive assessment of tumor oxygenation status may provide early insight into outcome following RT. In this regard, PAI can provide quantitative read-outs of tumor oxygenation without the need for radioactive tracers or contrast agents. This combined with the short acquisition times of PAI make it ideal for clinical imaging in a time-efficient and cost-effective manner. Therefore, in the present study, we sought to define the prognostic impact of PAI-based hemodynamic assessment in HNSCC.

While PAI is actively undergoing clinical evaluation for a variety of oncologic and non-oncologic applications, only a few studies have compared PAI measurements with conventional imaging methods such as MRI^22^,^23^. Good correlation between PAI and blood oxygenation level dependent MRI (BOLD-MRI) measurements has been recently reported^22^. However, the relationship between changes in BOLD contrast and tumor oxygenation is complex as signal change on BOLD-MRI is also sensitive to changes in blood flow and deoxy-hemoglobin content^24^,^25^. An alternative method that overcomes this limitation is OE-MRI that involves measurement of the change in R1 in response to hyperoxia^26^. Molecular oxygen is paramagnetic and shortens the T1 times of water containing dissolved oxygen, the measured change in R1 (=1/T1) is proportional to the tissue oxygen concentration^27^. Recent studies have highlighted the usefulness of OE-MRI as a biomarker of tumor oxygenation status in preclinical models and in patients^17^,^28^. We therefore examined the correlation between PAI and oxygen-enhanced MRI. We observed a significant increase in %SO_2_ and R1 in response to oxygen inhalation on PAI and MRI, respectively. The magnitude of signal increase at PAI (~15%) was higher than the change observed with OE-MRI imaging (~10%), highlighting the sensitivity of PAI. Whole tumor estimates did not reveal any correlation between the change in %SO_2_ and R1 of individual tumors following hyperoxia. Given the time interval between imaging sessions (~3 hours), discrepancies between PAI and OE-MRI in the tumors following oxygen challenge could be reflective of temporal fluctuations in tumor blood flow and oxygenation. Studies in experimental tumor models have reported frequent changes in perfusion and micro regional oxygenation^29^,^30^. It is therefore important to recognize the contribution of intermittent or transient changes in tumor perfusion and oxygenation on PAI and OE-MRI readouts. Consistent with these observations, our imaging data revealed considerable spatial heterogeneity in tumor oxygenation. Co-registration of PAI and OE-MRI data with *ex-vivo* histology demonstrated good spatial correlation in a majority of tumors. The limited correlation in some of the tumors could be attributed to differences in signal thresholding between PAI and MRI datasets and the lack of fiducial markers to accurately co-register imaging and histology data. ROI analysis showed a significant correlation between PAI and MRI data. A positive correlation between the PAI signal at 850 nm following hyperoxia and % viable tumor on histology was also observed. Collectively, our results demonstrate the sensitivity of PAI in detecting changes in tumor oxygenation and the ability of PAI to map inter- and intra-tumoral heterogeneity in oxygenation.

We then assessed the hemodynamic response of HNSCC to RT using PAI. Baseline and treatment-induced changes in imaging parameters of hemodynamics (oxygen saturation, hemoglobin concentration) were analyzed and correlated with outcome of individual tumors (animals) to define the prognostic importance of hemodynamic response assessment in HNSCC. We observed an early (24 h) increase in tumor oxygen saturation following RT on PAI examination in tumors that showed favorable prognosis. Similar observations on early oxygenation change following RT have been observed using non-invasive MRI and optical spectroscopic methods^31^,^32^,^33^. Cao *et al.* observed that early increase in blood volume of head and neck tumors detected by dynamic contrast-enhanced MRI correlated with tumor control following RT^31^. Using diffuse reflectance spectroscopy, Vishwanath *et al.*, have reported increased oxygen saturation in murine head and neck tumor xenografts starting at 5 days post RT that correlated with long-term tumor control^32^. An increase in oxyhemoglobin concentration has also been reported in breast tumors 24 h after chemotherapy on diffuse optical spectroscopic examination^33^. While the precise mechanism(s) behind these observations is unclear, possible explanations include (i) a decrease in cellular metabolism and oxygen consumption in tumors following RT induced cell death, (ii) increased tumor perfusion as a result of an acute inflammatory response triggered by RT-induced cellular damage^33^,^34^,^35^. These explanations are supported by observations in the published literature. Olive studied changes in SCC IVV tumor oxygenation after RT, and showed that increases in tumor oxygenation were related to increases in tumor perfusion in addition to decreased tumor oxygen consumption^35^. The observed increase in tumor perfusion likely results from the reduction in cell density and tumor interstitial fluid pressure^36^. In cervical cancer patients, Lyng *et al.* observed that tumors that showed increased tumor pO_2_ levels following RT also exhibited increased apoptotic frequency and consequently a decrease in tumor cell density^37^. In contrast, patients that had decreased tumor pO_2_ levels had no significant increase in apoptotic frequency and therefore a smaller decrease in cell density. Collectively, our results suggest that tumor oxygenation assessment by PAI can provide early insight into the treatment outcome following RT in HNSCC. Such insight could assist in patient selection and design of adjuvant therapies (e.g. radiosensitizers, oxygen-modifiers) to maximize therapeutic benefit.

Our studies also demonstrate the potential utility of PAI for monitoring radiation-induced salivary gland injury *in vivo*. In head and neck cancer patients, radiation-induced salivary gland injury results in xerostomia (severe dry mouth) that lasts for months or even years after treatment^18^,^38^. The biological response of salivary glands to RT includes parenchymal loss, acinar atrophy, lysis and fibrosis of glandular tissue^18^,^19^,^38^. Our PAI studies in mice revealed a reduction in the hemodynamic response of salivary glands to gustatory stimulation 24 h after RT (15 Gy) suggestive of early radiation-induced vascular injury of salivary glands. This is consistent with a previous observation by Xu *et al.*, in which evidence of endothelial damage was observed within 4–24 h following a single dose of RT^39^. Clinical assessment of xerostomia involves patient-based or physician-based grading systems that can be subjective and prone to variability^40^. Conventional scintigraphy methods^41^ allow for objective evaluation of salivary gland dysfunction but require the use of radioisotopes and are therefore not ideal for longitudinal assessment in patients. Our results suggest that PAI of salivary glands could be useful as a non-invasive alternative to repetitive scintigraphy or sialography. PAI examination is a quick, low-risk procedure that is relatively easy to perform and is therefore ideal for rapid, bedside examination. Given the anatomic proximity of head and neck tumors and normal tissue target of radiation (salivary glands), PAI could potentially allow for monitoring tumor response to RT while simultaneously assessing radiation injury of salivary glands. Given the coupling between blood flow and salivary secretion^42^ and the vascular effects of RT, assessment of salivary gland hemodynamics could be mechanistically informative. Early assessment of functional glandular changes in response to RT could also determine the course of management in these patients. Successful development and application of PAI for salivary gland imaging could potentially guide the study design and monitor the efficacy of agents that are aimed at protecting or salivary gland function.

Limitations of our study need to be recognized. Although PDX models are clinically relevant due to their ability to capture biological and molecular heterogeneity seen in human tumors, the need for immunocompromised animals does not permit investigation into the role of immune mechanisms in tumor response to RT. In addition, we performed studies using subcutaneous tumor models that do not reflect the true microenvironment of these tumors. For our salivary gland studies, we did not correlate our imaging changes with histologic assessment of salivary gland tissue. This would be important to understand the mechanism(s) that contribute to the loss of hemodynamic response following RT. Another limitation is that tumor and salivary gland studies were performed in separate cohorts of animals. Future studies should focus on using orthotopic HNSCC models in immunocompetent mice where changes in tumor and salivary gland oxygenation can be monitored simultaneously using PAI. In these studies, correlative immunostaining of tissues for markers of vascular function (CD31/lectin) and maturation (smooth muscle actin) would help understand the impact of vessel maturation on oxygen saturation and response to RT. And finally, while depth may not be a concern for imaging a superficial organ such as the salivary glands, it is an important consideration that influences PAI of tumors. Recent studies conducted in tissue-mimicking phantoms allowed us to detect up to 2.5 cm depth (Seshadri, unpublished observations). With development of next-generation PAI probes and improvements in light delivery techniques and signal processing methods, this could be further improved. Future studies should address these issues to fully realize the clinical potential of this imaging method in head and neck oncology.

## Materials and Methods

### Animal models

Experimental studies were performed using two PDX HNSCC models established in 6–8 wk old female severe combined immunodeficient (SCID) mice. The procedures for establishing PDX models in mice have been previously described^43^,^44^. Tumors were implanted subcutaneously on the flank or hind leg for experimental imaging and therapy studies. Salivary gland RT studies were performed in 6–8 wk old naïve male athymic nude mice (NCr-nu/nu) purchased from Harlan Sprague-Dawley (Indianapolis, Ind). Animals were housed in microisolator cages, provided standard chow/water and maintained on 12 h light/dark cycles in a HEPA-filtered environment. All experimental studies were performed in accordance with protocols approved by the Institutional Animal Care and Use Committee at Roswell Park Cancer Institute (RPCI).

### PAI with co-registered US

Experimental PAI with co-registered US was performed using a 21 MHz linear-array transducer system (Vevo® LAZR; VisualSonics Inc., Toronto). For combined PAI and OE-MRI studies tumor-bearing mice were anesthetized using ketamine:xylazine mixture (10 mg/ml: 1 mg/ml) administered intraperitoneally (i.p.) at a dose of 10 mg/kg and secured on a heated platform. Animals underwent PAI examination first and were allowed to recover (~3 hours) prior to OE-MRI to avoid potential complications relating to prolonged anesthesia for imaging. For all other PAI studies animals were anesthetized using 2.5% Isoflurane (Benson Medical Industries, Markham, ON, Canada). Three dimensional (3D) B-mode ultrasound images were acquired to obtain tumor volume measurements. Multispectral PAI was performed to obtain measurements of oxygen saturation (SO_2_) and hemoglobin (Hbt) as previously described^22^. Oxygen enhanced PAI was performed on a single central slice of the tumor. Animals were imaged during room air breathing (2 min) before switching to 100% oxygen breathing (6 mins) and subsequently returning to room air (6 mins). Continuous PA images were acquired throughout this procedure. For RT and CRT studies, 3D PAI datasets (9 mm) were also acquired. PAI was performed before (baseline) and 24 h following RT. The photoacoustic parameters used were; Transducer: LZ-250, Depth: 20.00 mm, Width: 23.04 mm, Wavelength: 750/850 nm, Threshold HbT: 17, acquisition: SO2/Hbt. Time gain compensation (TGC) was applied during PDX studies to adjust for signal loss at increased depths. For salivary gland imaging, PAI datasets were acquired before and following gustatory stimulation using 10% citric acid applied to the dorsum of the tongue. Following completion of imaging, animals were removed from the imaging platform and monitored to ensure full recovery. All imaging datasets were analyzed using the Vevo LAB (Ver 1.7.2) workstation software. PAI based measurements of oxygen saturation were calculated using the two-wavelength approach (750/850 nm) based on a previously reported algorithm^10^,^22^. For oxygen enhanced imaging studies and salivary gland studies, %SO_2 Average_ values, which represent the average oxygen saturation values of pixels with a %SO_2_ estimate are reported. For RT and CRT studies, %SO_2 Total_, which represents the average oxygen saturation of all pixels including those with a zero/void estimate are reported. These values are reported for the RT studies since they are more representative of oxygen saturation levels within the whole tumor, while %SO_2 Avg_ is representative of the degree of blood oxygenation and therefore was used to study changes in blood oxygenation in our OE-MRI and salivary gland studies. Analysis of oxygen enhanced imaging studies was performed by tracing a region of interest (ROI) for a single, central slice of the tumor. For oxygen enhanced imaging studies, the relative percent change in %SO_2_ when transitioning from air to oxygen and oxygen back to air was calculated using the following formula [(%SO_2 post_ – %SO_2 pre_)/%SO_2 pre_]*100. Analysis of RT and CRT studies was performed by tracing a 3D ROI for a 9 mm section of the tumor. Calculation of salivary gland %SO_2 Avg_ levels was performed by tracing an ROI for a single, central slice of the salivary gland. The relative percent change in salivary gland %SO_2_ following stimulation was calculated using the formula [(%SO_2 post-stim_ – %SO_2 pre-stim_)/%SO_2 pre-stim_]*100. Color maps representing %SO_2_ were displayed using a color look-up table superimposed on corresponding ultrasound images. Statistical analysis and graphical display of data was performed using Graphpad® software (Version 6.00 for Windows, GraphPad Software, SanDiego California USA, http://www.graphpad.com). Error bars represent the standard error of the mean (SEM).

### Magnetic resonance imaging

MR images were acquired using a 4.7 T/33-cm horizontal bore magnet (GE NMR Instruments, Fremont, CA) incorporating AVANCE digital electronics (Bruker Biospec with ParaVision 2.1; Bruker Medical, Billerica, MA). Mice were anesthetized using ketamine:xylazine mixture (10 mg/ml: 1 mg/ml) administered intraperitoneally (i.p.) at a dose of 10 mg/kg. Animals were secured in a carrier tube and positioned in the scanner using an MR-compatible mouse sled equipped with temperature and respiratory sensors (Dazai Research Instruments, Toronto, Canada). Animal body temperature was maintained at 32 °C using an air heater system (SA Instruments Inc., Stony Brook, NY) with feedback from electrothermal sensors in the sled. Data acquisition consisted of localizer images, T2-weighted (T2W) and T1-weighted (T1W) spin echo (SE) images. T2-weighted spin echo images were acquired with the following parameters: TE_eff_ = 41.0 ms; TR = 2500 ms; field of view = 3.2 cm × 3.2 cm; matrix size = 256 × 192; slice thickness = 1.00 mm; interslice distance = 1.25 mm, 21 slices, number of averages (NEX) = 4. T1-mapping of tumors was performed during air and oxygen breathing using a saturation recovery fast spin echo (T1-FSE) sequence as previously described by us^44^. T1-weighted spin echo images were acquired with the following parameters: TE_eff_ = 25.0 ms; TR = 6000, 3000, 1500, 750, 500, 360.34 ms; RARE/Echoes = 4/4; 3.2 cm × 3.2 cm; matrix size = 128 × 96; slice thickness = 1.00 mm; interslice distance = 1.25 mm; 5 slices, number of average (NEX) = 1. Extreme care was taken to maintain consistent animal position between PAI and MRI to help achieve accurate spatial correlation between imaging modalities. Anatomical landmarks of the skin line on the top of the tumor and necrotic regions were used to spatially co-register PA and MR images. Post processing of the raw MR data was performed using the medical imaging software, Analyze PC (Analyze PC, Version 7.0, Biomedical Imaging Resource, Mayo Clinic, Rochester, MN). Parametric maps of tumor relaxation rate (R1 = 1/T1) were calculated in MATLAB (Version 2013a; Mathworks, Natick, MA)^42^. Color maps representing the change in R1 before and after oxygen breathing were displayed using a color LUT superimposed on corresponding T2-weighted anatomical images.

### Histopathology

Following comparative PA and OE-MR imaging, tumors were removed and fixed in 10% formalin to perform histopathology. Skin was left on top of the tumor to assist in the spatial co-registration of histology and imaging datasets. A black line was traced on top of the skin corresponding to the imaging plane to reference during sectioning. Slides were stained with Harris haematoxylin (Poly Scientific, Bay Shore, NY) as described previously^41^. Glass slides containing stained tumor sections were scanned and digitized using the ScanScopeXT system (Aperio Technologies, Vista, CA).

### Chemotherapy and radiation treatment

For CRT studies, mice were given 1 mg cetuximab i.p. (Erbitux, ImClone LLC, New York, NY) immediately following baseline imaging, with a single radiation dose of 7.5 Gy given 24 h after cetuximab treatment. Mice in the RT alone and salivary gland studies were given a single radiation dose of 15 Gy. Mice receiving RT were anesthetized with 2.5% isoflurane, placed on a heated platform, and monitored with a closed circuit surveillance camera system. Irradiation was performed using the Philips RT 250 Orthovoltage X-ray unit (Philips Medical Systems, Andover, MA) running at 75 kV/17.7 mA with a 2 mm aluminum filter and 1 × 2 cm applicator cone. The dose rate of the unit at these settings was ~0.68 Gy/min. Radiation was delivered through an axial beam directed to the tumor or neck of mice (submandibular-sublingual salivary gland complex). A lead shield designed to protect the tissues (other than the target volume) from exposure to radiation was utilized. Animals were observed to ensure complete recovery following treatment.

### Therapeutic response assessment

Animals were monitored for 2 weeks following treatment to assess long-term response to therapy. Animals were euthanized if animals exhibit greater than 20% loss in body weight or if signs of morbidity appeared.

### Sample sizes and statistical considerations

Comparative evaluation of tumor hemodynamics using PAI and MRI was performed in subcutaneous PDX HNSCC xenografts established in female SCID mice (n = 8). Spatial co-registration of PAI and MRI datasets with *ex-vivo* histology (H&E) was performed in all 8 tumors following imaging. However, alignment of PA and MR images was not successful in one of the tumors (m4). Response of HNSCC to CRT (n = 6) or RT (n = 8) was studied using PAI in two different PDX cohorts. One of the tumors that underwent CRT was identified as a statistical outlier (Grubb’s test) and excluded from analysis. The reported PAI values for CRT response therefore consist of 5 tumors. Pearson correlation was performed to compare change in oxygen saturation at 24 h and change in tumor volume at 2 weeks. Assessment of radiation-induced salivary gland injury was performed in mice before and 24 h after 15 Gy RT (n = 4). Statistical analyses and graphical display of data were performed using Graphpad Prism (GraphPad Software, San Diego, CA). Mean ± standard error is reported in all bar graphs. Two-tailed paired student t-tests were used to compare pre and post oxygen PAI and MRI datasets in addition to salivary gland stimulation studies. Statistical comparison of baseline and post CRT/RT tumor %SO_2_ levels was performed using the non-parametric Mann-Whitney test. Pearson’s correlation tests were performed to compare PAI, MRI and histology data. P-values <0.05 were considered significant.

## Additional Information

**How to cite this article**: Rich, L. J. and Seshadri, M. Photoacoustic monitoring of tumor and normal tissue response to radiation. *Sci. Rep.* **6**, 21237; doi: 10.1038/srep21237 (2016).

## Supplementary Material

Supplementary Information
