# Supplementary material for: Photoacoustic monitoring of tumor and normal tissue response to radiation
Source: Sci Rep. 2016 Feb 17;6:21237. doi: 10.1038/srep21237 (PMC4756329; doi:10.1038/srep21237)
Supplement: Supplementary Information [file srep21237-s1.pdf]

Photoacoustic monitoring of tumor and normal tissue response to radiation

Laurie J. Rich[a,b] and Mukund Seshadri[a,b,c1]

Departments of [a]Molecular and Cellular Biophysics and Biochemistry, [b]Pharmacology and Therapeutics, [c] Oral Medicine/Head and Neck Surgery, Roswell Park Cancer Institute, Buffalo, NY 14263

Supplementary Figures

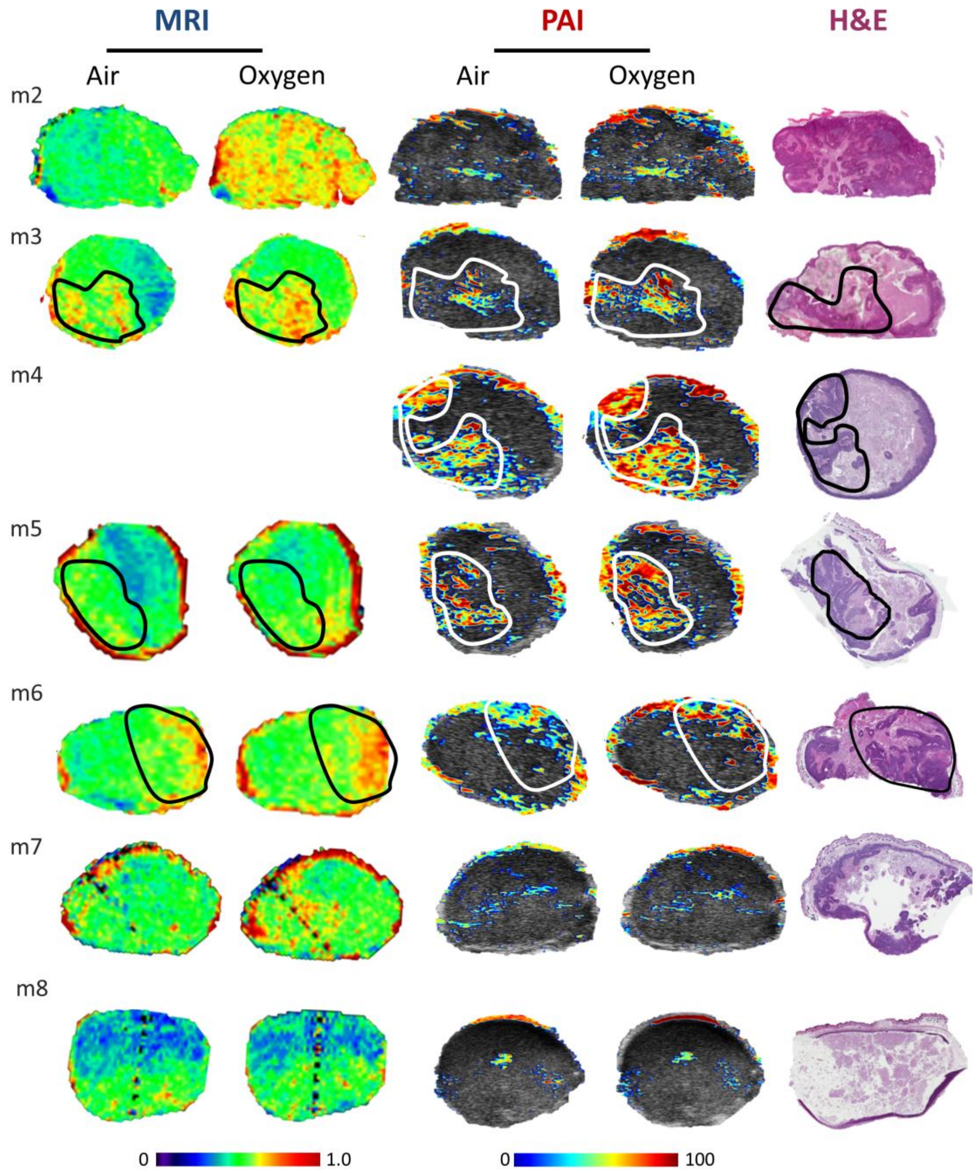

Supplementary Figure S1. Spatial correlation between PAI, MRI and ex-vivo histology. Ex-vivo histology of the remaining 7 tumors that underwent PAI and MRI are shown. Spatial alignment between MRI and PAI was not possible in one of the tumors (m4). While the degree of spatial correlation was limited in some of the tumors (m2, m5), we observed good correlation between PAI and histology in 6/8 tumors (m1, m3, m4, m5, m7, m8) and between PAI with OE-MRI in 5/7 tumors (m1, m3, m6, m7, m8). Tumors m3-m6 showed enhancement only in sub-regions of the tumor (black and white outlines) that corresponded to viable areas on histology. Tumor m5 showed no enhancement in the viable area of tissue observed with H&E, but PAI showed strong enhancement in this region. Tumors m7 and m8 showed limited enhancement on OE-MRI and PAI, and H&E revealed that these tumors had considerable levels of necrosis.

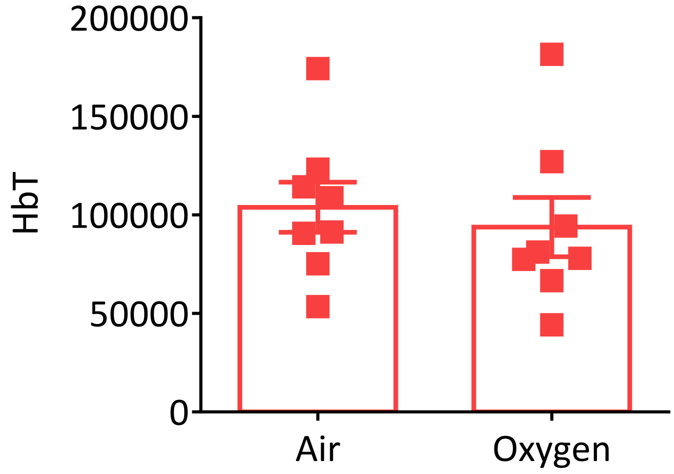

Supplementary Figure S2. Values of total hemoglobin concentration (HbT) before and after oxygen challenge are shown. Tumor-bearing mice were exposed to a regimen of room air for 2 minutes followed by inhalation of 100% of oxygen (hyperoxia) for 6 minutes and subsequently returned to room air. No significant change in HbT was observed in response to oxygen inhalation.

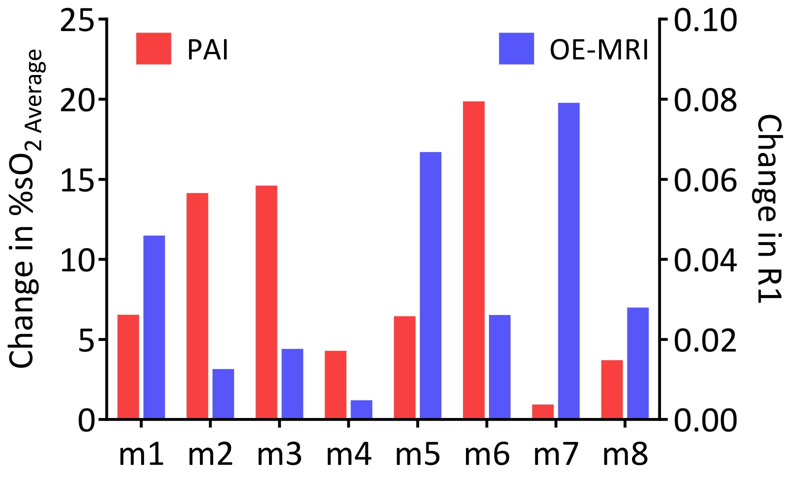

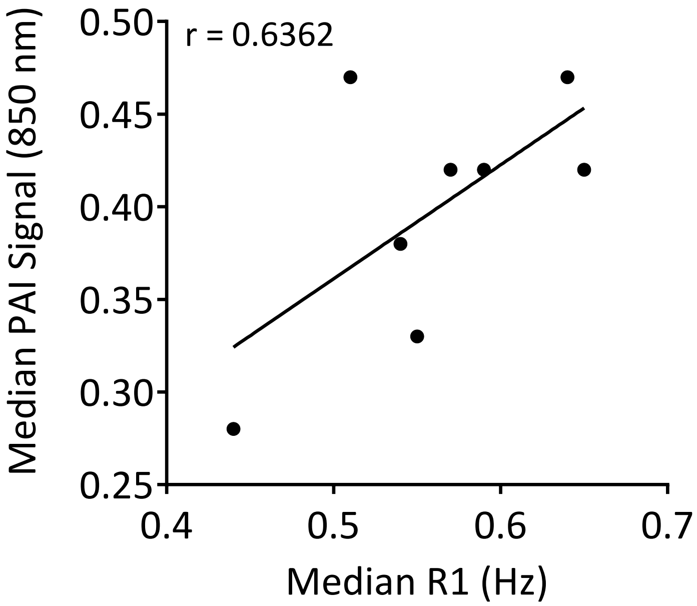

Supplementary Figure S3. *Top*: Bar graphs showing change in $\%sO_{2\ average}$ (red bars) and R1 (blue bars) of individual tumors following hyperoxia. Values represent measurements made over the whole tumor. *Bottom:* Correlation between median PAI signal (850 nm) and median R1 values for individual tumors (black circles) ($p = 0.08$).

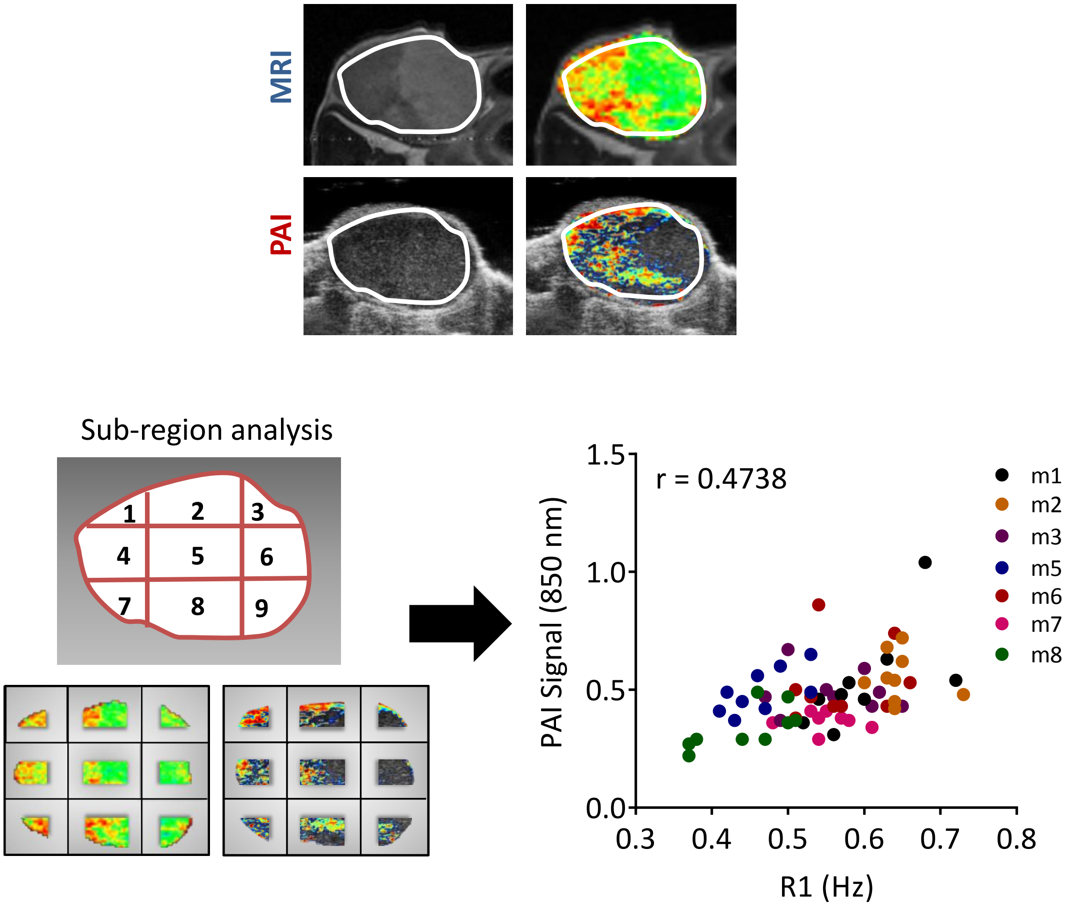

Supplementary Figure S4. Region of interest analysis was performed for spatial correlation of post-oxygen PAI and MRI datasets. Correlation analysis of 9 matching ROIs traced within individual tumors revealed a significant correlation (r = 0.4738, p = <0.0001) between PAI and MRI.

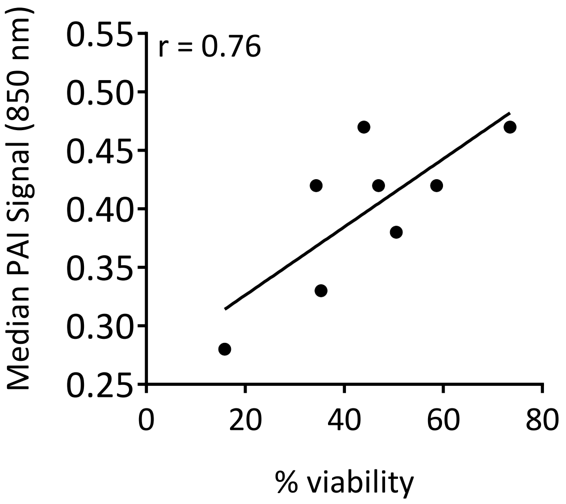

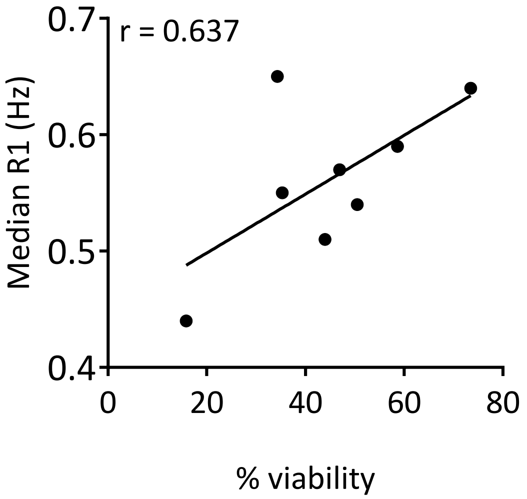

Figure S5. Correlation between median PAI signal (top; p = 0.02) and median R1 (bottom; p = 0.08) following hyperoxia with estimates of tumor viability from ex-vivo histology.

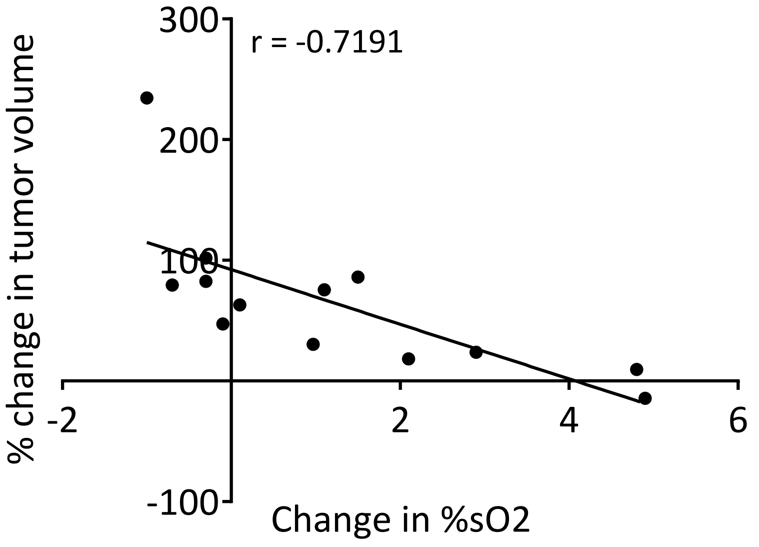

Figure S6. Change in oxygen saturation estimates measured at 24 hours compared to tumor volume change over a two week day period following RT or CRT PDX-HNSCC. A significant correlation ($p = 0.005$) was observed between the early oxygenation change detected by PAI and the degree of tumor growth inhibition observed at 2 weeks.

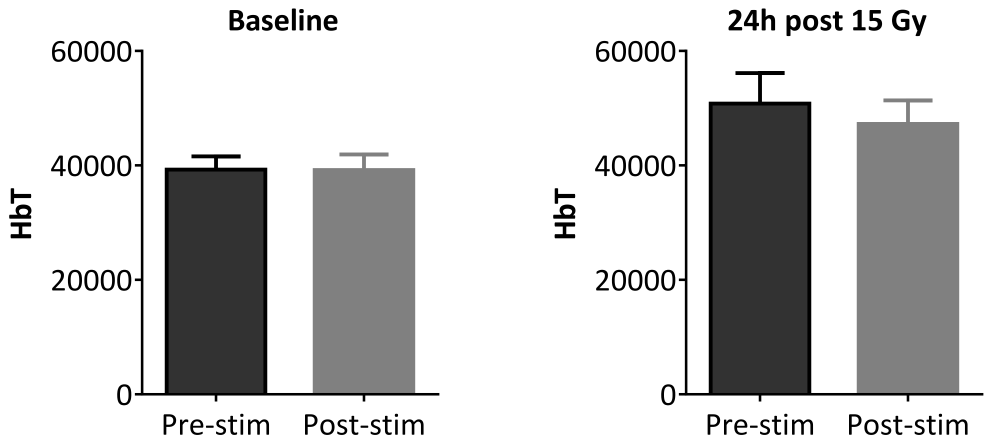

Figure S7. Change in hemoglobin concentration (HbT) of naïve mouse salivary glands before and after gustatory stimulation. Values were obtained at baseline and 24 h post RT. No change in HbT was seen following RT compared to baseline estimates.

SUPPLEMENTARY METHODS

Region of interest analysis

Region of interest (ROI) analysis was performed on post oxygen PAI and MRI datasets using Analyze PC (Analyze PC, Version 7.0, Biomedical Imaging Resource, Mayo Clinic, Rochester, MN). For comparison of OE-MR and PA images, the 850 nm PA image was selected since oxygenated hemoglobin is the dominant optical species at this wavelength. The 3-D voxel registration tool was used to spatially align PA and MR images. An outline of the tumor was traced simultaneously for a single, matching slice of both datasets and divided into 9 ROI (Fig. S4), and values of PAI signal and R1 extracted for correlation analysis. This was performed for all tumors (7 tumors x 9 regions per tumor = 63 regions) excluded m4 where spatial co-registration was not possible.
